# Enhanced cytotoxicity of a *Pseudomonas* Exotoxin A based immunotoxin against prostate cancer by addition of the endosomal escape enhancer SO1861

**DOI:** 10.3389/fphar.2023.1211824

**Published:** 2023-07-06

**Authors:** Anie P. Masilamani, Nathalie Huber, Constanze Nagl, Viviane Dettmer-Monaco, Gianni Monaco, Isis Wolf, Susanne Schultze-Seemann, Sanaz Taromi, Christian Gratzke, Hendrik Fuchs, Philipp Wolf

**Affiliations:** ^1^ Department of Urology, Medical Center—University of Freiburg, Freiburg, Germany; ^2^ Faculty of Medicine, University of Freiburg, Freiburg, Germany; ^3^ Institute for Transfusion Medicine and Gene Therapy, Medical Center—University of Freiburg, Freiburg, Germany; ^4^ Center for Chronic Immunodeficiency, Medical Center—University of Freiburg, Freiburg, Germany; ^5^ Institute of Neuropathology, Medical Center—University of Freiburg, Freiburg, Germany; ^6^ Faculty for Biology, University of Freiburg, Freiburg, Germany; ^7^ Department of Medicine I, Medical Center—University of Freiburg, Freiburg, Germany; ^8^ Faculty of Medical and Life Sciences, University Furtwangen, VS-Schwenningen, Germany; ^9^ Institute of Diagnostic Laboratory Medicine, Clinical Chemistry and Pathobiochemistry, Charité—Universitätsmedizin Berlin, Corporate Member of Freie Universität Berlin and Humboldt-Universität zu Berlin, Berlin, Germany

**Keywords:** prostate cancer, immunotoxin, *Pseudomonas* exotoxin A, glycosylated triterpenoid, endosomal escape, SO1861

## Abstract

Immunotoxins consist of an antibody or antibody fragment that binds to a specific cell surface structure and a cytotoxic domain that kills the cell after cytosolic uptake. *Pseudomonas* Exotoxin A (PE) based immunotoxins directed against a variety of tumor entities have successfully entered the clinic. PE possesses a KDEL-like motif (REDLK) that enables the toxin to travel from sorting endosomes via the KDEL-receptor pathway to the endoplasmic reticulum (ER), from where it is transported into the cytosol. There, it ADP-ribosylates the eukaryotic elongation factor 2, resulting in ribosome inhibition and finally apoptosis. One major problem of immunotoxins is their lysosomal degradation causing the need for much more immunotoxin molecules than finally required for induction of cell death. The resulting dose limitations and substantially increased side effects require new strategies to achieve improved cytosolic uptake. Here we generated an immunotoxin consisting of a humanized single chain variable fragment (scFv) targeting the prostate specific membrane antigen (PSMA) and the de-immunized PE variant PE24mut. This immunotoxin, hD7-1(VL-VH)-PE24mut, showed high and specific cytotoxicity in PSMA-expressing prostate cancer cells. We deleted the REDLK sequence to prevent transport to the ER and achieve endosomal entrapment. The cytotoxicity of this immunotoxin, hD7-1(VL-VH)-PE24mutΔREDLK, was greatly reduced. To restore activity, we added the endosomal escape enhancer SO1861 and observed an up to 190,000-fold enhanced cytotoxicity corresponding to a 57-fold enhancement compared to the initial immunotoxin with the REDLK sequence. A biodistribution study with different routes of administration clearly showed that the subcutaneous injection of hD7-1(VL-VH)-PE24mutΔREDLK in mice resulted in the highest tumor uptake. Treatment of mice bearing prostate tumors with a combination of hD7-1(VL-VH)-PE24mutΔREDLK plus SO1861 resulted in inhibition of tumor growth and enhanced overall survival compared to the monotherapies. The endosomal entrapment of non-toxic anti-PSMA immunotoxins followed by enhanced endosomal escape by SO1861 provides new therapeutic options in the future management of prostate cancer.

## 1 Introduction

Prostate cancer is listed as the second most common cancer in men worldwide. More than 1.4 million patients are diagnosed with this disease and more than 375,000 die from it every year (Sung et al., 2021). A timely diagnosis is important because early tumor stages can be successfully treated by surgery or radiation. Advanced stages can be managed by combination treatments including chemo-/hormonal treatment or radiation. These treatments, however, are no longer curative. Therefore new therapeutic options are urgently needed (Teo et al., 2019).

Immunotoxins are recombinant or chemically linked proteins consisting of a ligand (antibody or antibody fragment), which binds to a specific cell surface structure and a cytotoxic domain of bacterial, plant or synthetic origin, which is able to kill the cell after internalization (Wolf, 2021).

One prominent toxin for the construction of immunotoxins is *Pseudomonas* Exotoxin A (PE), a virulence factor of the bacterium *Pseudomonas aeruginosa.* PE is a 613 a protein with an N-terminal part, called domain Ia (aa 1–252), for binding to the widespread receptor CD91 (α2MR/LRP) on eukaryotic host cells. This domain can be exchanged for an antibody or antibody fragment targeting an antigen on the surface of tumor cells so that a tumor-specific immunotoxin is formed. The C-terminal part of PE has a molecular mass of about 40 kDa and is therefore also called PE40. It consists of domain II (aa 253–364) for the translocation across cell membranes including a furin-cleavable site (FCS, aa 274–280) and of the domains Ib (aa 365–404) and III (aa 405–613) that form the cytotoxic subunit with ADP-ribosyltransferase activity. PE is able to ADP-ribosylate the eukaryotic elongation factor-2 (eEF-2) on ribosomes, which leads to a protein biosynthesis inhibition in the cell and finally to apoptosis (Wolf and Elsasser-Beile, 2009; Michalska and Wolf, 2015). The last five amino acids at the C-terminus of the cytotoxic subunit of PE (aa 609–613, REDLK) form a KDEL-like motif for trafficking via the KDEL receptor-mediated pathway in eukaryotic cells (Hessler and Kreitman, 1997).

PE-based immunotoxins act as follows: First, the C-terminal lysine (K) is proteolytically cleaved by plasma carboxypeptidases so that the motif REDL is formed (Hessler and Kreitman, 1997). After antigen binding, PE-based immunotoxins are internalized into the tumor cells and the cytotoxic part of PE is cleaved from the binding domain by furin within the acidic endosomal environment. It can bind to KDEL receptors with help of the REDL motif and travels retrogradely via the KDEL receptor-mediated pathway from the late endosomes through the trans-Golgi network into the endoplasmic reticulum (ER). Then it is secreted from the ER into the cytosol, where it can display its cytotoxicity by ADP-ribosylation of eEF-2 and kill the target cell (Wolf and Elsasser-Beile, 2009; Michalska and Wolf, 2015).

Truncated PE variants lacking the binding domain Ia where shown to be not cytotoxic, because they are not able to bind and to internalize into target cells (Hwang et al., 1987; Xing et al., 2021). PE-based immunotoxins can be generated by exchange of the binding domain against antibodies, antibody fragments or ligands targeting tumor-associated antigens and receptor-mediated internalization into the endolysosomal compartment can be used for effective trafficking of the PE domain into the cytosol (Wolf and Elsasser-Beile, 2009). In the past, different PE-based immunotoxins against prostate cancer targeting the epidermal growth factor receptor family, interleukins, or the prostate specific membrane antigen (PSMA) were developed and tested preclinically (Debinski and Pastan, 1994; Maini et al., 1997; Kawakami et al., 2001; Husain et al., 2003; Baiz et al., 2013; Niesen et al., 2015; Meng et al., 2017; Fischer et al., 2020; Xing et al., 2021). We have generated immunotoxins consisting of the murine single chain variable fragment (scFv) D7 (Michalska et al., 2016; Michalska et al., 2018) or the humanized variant thereof, called hD7-1 (Masilamani et al., 2020), that bind to PSMA as binding domains. PE40 or the de-immunized PE variant PE24mut with only 24 kDa in size were used as cytotoxic domains. The latter lacks domain II with exception of the FCS and contains alanine mutations in seven immunodominant B-cell epitopes (R457A, R458A, D463A, R467A, R490A, R505A, R538A) in domain III (Liu et al., 2012). Immunotoxins with the PE24mut domain are less immunogenic in patients and lower the risk to develop the vascular leak syndrome (VLS) (Weldon et al., 2013; Mazor and Pastan, 2020), a main off-target toxicity of PE-based immunotoxins in the clinic (Alewine et al., 2020).

hD7-1(VL-VH)-PE40 showed a high and specific cytotoxicity against PSMA expressing prostate cancer cells and elicited synergistic antitumor activity in combination with the BH3 mimetic ABT-737 by collaborative downregulation of pro-survival Bcl-2 family proteins (Masilamani et al., 2020). The immunotoxin hD7-1(VL-VH)-PE24mut retained PSMA specificity and high cytotoxicity on different prostate cancer cells with IC_50_ values in the picomolar range with a 3.3- to 4.6-fold reduced cytotoxicity compared to hD7-1(VL-VH)-PE40 (Michalska et al., 2018).

An important rate-limiting step in the use of immunotoxins is an insufficient endosomal escape after internalization, which inevitably leads to lysosomal degradation and thereby inactivation of the immunotoxins (Lönn et al., 2016). Therefore, cells have to be incubated with much more immunotoxin molecules than finally required for induction of cell death resulting in dose limitations and substantially increased side effects.

To avoid this, a number of techniques to augment cytosolic uptake of protein-based targeted toxins have been developed during the last decades and include diverse chemicals, cell-penetrating or fusogenic peptides, and light-induced techniques (Fuchs et al., 2016). One family of molecules that enhance endosomal escape comprises glycosylated triterpenoids. These are plant secondary metabolites that can integrate into cell membranes without affecting integrity and can be taken up into endolysosomal membranes. It is thought that the glycosylated triterpenoids can associate with proteins that were internalized into endolysosomal compartments and mediate their release through the membrane into the cytosol (Fuchs et al., 2017). They were successfully used for the endosomal release of antibody drug conjugates, targeted toxins or immunotoxins containing the plant toxins dianthin and saporin, which resulted in enhanced cytotoxicity by several orders of magnitudes and enhanced antitumor activity *in vivo* (Holmes et al., 2015; von Mallinckrodt et al., 2014; Gilabert-Oriol et al., 2015; Gilabert-Oriol et al., 2016; Bhargava et al., 2017; Panjideh et al., 2022). For yet unknown reasons, glycosylated triterpenoids do not generally lead to a decisive increased cytosolic uptake of internalized proteins. For example, cytotoxicity of a targeted toxin consisting of EGF and the cytotoxic domain of PE was only 1.2-fold enhanced by addition of the glycosylated triterpenoid SA1641 (Weng et al., 2012a).

In the present study we initially tested the anti-PSMA immunotoxin hD7-1(VL-VH)-PE24mut in combination with the glycosylated triterpenoid SO1861, however, found no enhanced cytotoxicity. Surprisingly, the cytotoxicity of the non-toxic control immunotoxin hD7-1(VL-VH)-PE24mutΔREDLK lacking the C-terminal REDLK motif for trafficking via the KDEL-receptor mediated pathway, was strongly increased by the endosomal escape enhancer and even higher than that of the immunotoxin hD7-1(VL-VH)-PE24mut. Moreover, treatment of mice bearing prostate tumors with hD7-1(VL-VH)-PE24mutΔREDLK plus SO1861 led to significant inhibition of tumor growth and enhanced survival compared to monotherapies. Our study proves that endosomal entrapment of *per se* non-toxic PE-based immunotoxins followed by activation through endosomal escape is an effective new strategy for the future treatment of prostate cancer.

## 2 Materials and methods

### 2.1 Cell culture

PSMA-positive prostate cancer cell lines LNCaP and C4-2 were cultivated in RPMI 1640 medium and the PSMA-negative cell line PC3 (ATCC, Manassas, VA, United States) in F12 medium (Gibco, Invitrogen, Karlsruhe, Germany) containing 10% fetal calf serum (Biochrom, Berlin, Germany) and penicillin/streptomycin (100 U/mL, 100 mg/L) at 37°C and 5% CO_2_. Cell line identities were verified using short tandem repeat (STR) analysis (CLS GmbH, Eppelheim, Germany).

### 2.2 Preparation of SO1861

SO1861 was isolated from *Saponaria officinalis* L. as described (Gilabert-Oriol et al., 2016). Purity and identity were analyzed via LC/MS with an Agilent 6210 TOF LC/MS system. SO1861 was dissolved at a concentration of 200 μg/mL in distilled water, aliquoted and stored at −20°C. After thawing, SO1861 was incubated at 50°C for 10 min and was homogenized 30 times for 30 s each in an ultrasonic bath before use.

### 2.3 Generation of the immunotoxins

The immunotoxin hD7-1(VL-VH)-PE24mut, consisting of the humanized anti-PSMA scFv hD7-1 in VL-VH orientation and the de-immunized version of the cytotoxic domain of *Pseudomonas aeruginosa* Exotoxin A, called PE24mut (Liu et al., 2012), was cloned into the vector pHOG21 as described (Michalska et al., 2018). For the construction of hD7-1(VL-VH)-PE24mutΔREDLK, the cytotoxic domain with the deleted REDLK motif was synthesized by Geneart (Regensburg, Germany) and cloned C-terminally to the scFv in pHOG21. The immunotoxins were periplasmatically expressed in *E. coli* XL-1 blue bacteria and purified via immobilized metal affinity chromatography (IMAC) as published (Masilamani et al., 2020). Purified immunotoxins were analyzed by SDS PAGE and purity was calculated using a ChemiDoc MP Imaging System and the Image Lab Software (Bio-Rad, Hercules, California, United States). Immunotoxins were dialyzed against PBS and sterile filtered using a 0.2 μm protein filter. Protein content was determined with help of the Bradford Assay (Bio-Rad Laboratories, Feldkirchen, Germany). The immunotoxins were aliquoted and stored at −20°C until use.

For the biodistribution studies, hD7-1(VL-VH)-PE24mutΔREDLK was labeled with the fluorescence dye IR800CW according to the manufacturer’s instructions (LI-COR Biosciences, Bad Homburg, Germany).

### 2.4 Western Blot analyses

PSMA expression on the target cells was verified by Western Blot using the anti-PSMA mAb K7 (Elsässer-Beile et al., 2006) and horse radish peroxidase (HRP)-labeled polyclonal rabbit anti-mouse IgG (Dako, Hamburg, Germany) as detection antibodies. To analyze protein biosynthesis inhibition, target cells that were treated with hD7-1(VL-VH)-PE24mutΔREDLK and SO1861 for 24 or 48 h were incubated with 5 μg/mL puromycin (Tocris, Bio-Techne GmbH, Wiesbaden, Germany) for 15 min followed by lysis in radioimmunoprecipitation assay (RIPA) buffer (50 mM Tris-HCl, 150 mM NaCl, 1 mM EDTA, 0.5% NaDeoxycholate, 0.05% SDS, 1% Igepal). Anti-puromycin mouse mAb (Merck) and HRP conjugated rabbit anti-mouse Ab (Dako) were used for the detection of proteins by Western Blot that were translated at the time of lysate preparation. Apoptosis was detected in the lysates using Cas-3 mouse mAb (ECM Biosciences, Versailles, KY) and HRP conjugated rabbit anti-mouse polyclonal antibody (pAb) (Dako, Hamburg, Germany) as well as poly (ADP-ribose) polymerase (PARP) rabbit pAb (Cell Signaling Technology Europe) and HRP conjugated rabbit anti-mouse pAb (Dako). ß-actin rabbit pAb (Cell Signaling Technology Europe) and HRP conjugated goat anti-rabbit pAb (LI-COR Biosciences) were used for the detection of ß-actin as loading control. Western Blots were developed with an enhanced chemiluminescence (ECL) system and protein bands were detected and analyzed using the ChemiDoc MP Imaging System and the Image Lab Software (Bio-Rad Laboratories).

### 2.5 Phase contrast and fluorescence microscopy

Morphology of LNCaP cells was analyzed after treatment with 2.5 nM hD7-1(VL-VH)-PE24mutΔREDLK and 1.5 μg/mL SO1861 alone or in combination for 48 h. 25 μM of the pan-caspase inhibitor QVD-OPh was added to inhibit apoptosis.

For nuclear staining, cells were incubated with the immunotoxin and/or SO1861 and fixed with 2% paraformaldehyde solution for 30 min at RT. Then cells were washed with PBS and the intrinsic cell fluorescence was quenched by addition of 60 mm ammonium chloride, pH 7.4, for 20 min at RT. After repeated washing, cells were stained with 1% Hoechst 33342 (Molecular Probes, Eugene, Oregon, United States) and mounted with Vectashield Mounting Medium (Vector Laboratories Inc., Burlingame, CA). Phase-contrast and fluorescence images were recorded with help of a Zeiss AxioObserver Z.1 inverted microscope (Carl Zeiss Microscopy GmbH, Munich, Germany).

### 2.6 Flow cytometry

Cell binding of the immunotoxins was measured by flow cytometry as described previously (Michalska et al., 2016). Mouse anti-human c-myc mAb (BD Biosciences, Heidelberg, Germany) and goat anti-mouse Ig-R-PE Ab (Southern Biotech, Birmingham, AL, United States) were used as detection antibodies.

### 2.7 Cell viability assays

Cells were seeded at a concentration of 1.5×10^4^ cells/well into 96-well plates. After overnight incubation, cells were treated with SO1861 for 15 min followed by addition of immunotoxin. After 48 h, cell viability was determined with help of the WST-1 viability assay (Roche Diagnostics, Mannheim, Germany) according to the manufacturer’s instructions. Quantification of apoptotic and necrotic cells after treatment was analyzed using the Apoptosis and Necrosis Quantification Kit Plus, according to the manufacturer’s protocol (Biotium, Fremont, CA, United States).

### 2.8 *In vivo* experiments

Animal experiments were carried out in accordance with the German animal protection law and with permission from the responsible local authorities. Male SCID CB17/lcr-Prkdc^scid^/Crl mice (5–6 weeks old, 20–25 g, Janvier Labs, Saint-Berthevin, France) were kept under sterile and standardized environmental conditions.

For biodistribution studies, SCID mice (*n* = 2-3 per group) were subcutaneously injected with 1.5×10^6^ luciferase-transduced C4-2^luc+^ cells in 100 µL PBS mixed with 100 µL Matrigel^®^ (Corning, Kaiserslautern, Germany) into the right flank. Growing tumors were palpated and tumor diameters were measured in two axes using a caliper. Tumor volumes were calculated using the formula V = (d^2^×D)/2, where d was the smallest diameter and D the largest diameter of the tumor. For distribution studies, tumor bearing animals received 0.8 mg/kg hD7-1(VL-VH)-PE24mutΔREDLK-IR800CW intratumorally (i.t.), subcutaneously (s.c.), intravenously (i.v.) or intraperitoneally (i.p.). Control mice remained untreated. Tumor uptake of the targeted toxin in the following 48 h was detected by *in vivo* bioluminescence imaging (BLI, to detect the tumors) in combination with fluorescence imaging (FLI, to detect the immunotoxin). For this, 150 mg/kg of luciferin (BioSynth AG) were injected i.p. into the animals and BLI/FLI was done 10–30 min after injection under anesthesia using the *In vivo* Imaging System (IVIS) 200 (Xenogen VivoVision).

For therapy, mice with tumors of approx. 35–100 mm^3^ volume were divided into treatment groups of 8–10 animals each (day 1 of treatment). The first group received 30 µg SO1861 injected s.c. into the nuchal fold. The second and third treatment groups were injected with 0.8 mg/kg hD7-1(VLVH)-PE24mut or 0.8 mg/kg hD7-1(VLVH)-PE24mutΔREDLK s.c. next to the tumor. For combination, animals were injected with hD7-1(VLVH)-PE24mutΔREDLK followed by s.c. injection of 30 µg SO1861 1 hour later. Another control group remained untreated. Mice received their respective therapies on days 1, 3, 8, 10, 15, 17 of treatment and were observed until end of treatment on day 75. During therapy tumors were palpated and tumor volume was calculated as described above. Animals were euthanized if any of the following terminal criteria were met: largest tumor diameter >15 mm, ulcerating tumor, invasive tumor growth, weight loss >20% over two consecutive days.

### 2.9 Statistics

Binding affinity (K_d_) of the immunotoxins, defined as the half-maximal saturation concentration on prostate cancer cells in flow cytometry was calculated using the software GraphPad Prism version 8.4.3 (San Diego, CA, United States). The IC_50_ values, defined as the targeted toxin/SO1861 concentrations that lead to a reduction in cell viability by 50%, were estimated by non-linear regression [log (immunotoxin) vs. response (three parameters)] and significance in cytotoxicity was determined by unpaired, parametric Student’s *t-*test with Welch’s correction using GraphPad Prism. Enhancement of cytotoxicity (EOC) of the immunotoxins by SO1861 was calculated with help of the formula EOC = IC_50_ (Immunotoxin)/IC_50_ (Immunotoxin + SO1861). The Bliss Independence Model was used to determine the Combination Index (CI) as a measure of synergism in cytotoxicity (Zhao et al., 2014). Data from nuclear staining were analyzed using unpaired, parametric Student’s *t-*test with Welch’s correction. Tumor growth inhibition was determined by calculating the time-adjusted Area Under the Curve (AUC) (software GraphPad Prism version 8.2.1) as described by Duan et al. (Duan et al., 2012). Values between measurements were interpolated using the “natural” method of the spline function (https://www.R-project.org/). Differences in AUC were analyzed using the student’s *t-*test (unpaired, parametric with Welch’s correction). Overall survival was examined by Kaplan Meier survival analysis and log-rank test using GraphPad Prism. Tumor regression to growth ratio as the sum of tumor mass loss and tumor mass gain over all tumors within each treatment group was calculated as described (Panjideh et al., 2022). If one tumor completely disappears during treatment and another one with the same volume doubles in size, then the ratio for these two tumors is 1.0. This means that the higher the ratio the better the success of the treatment.

## 3 Results

**FIGURE 1:**
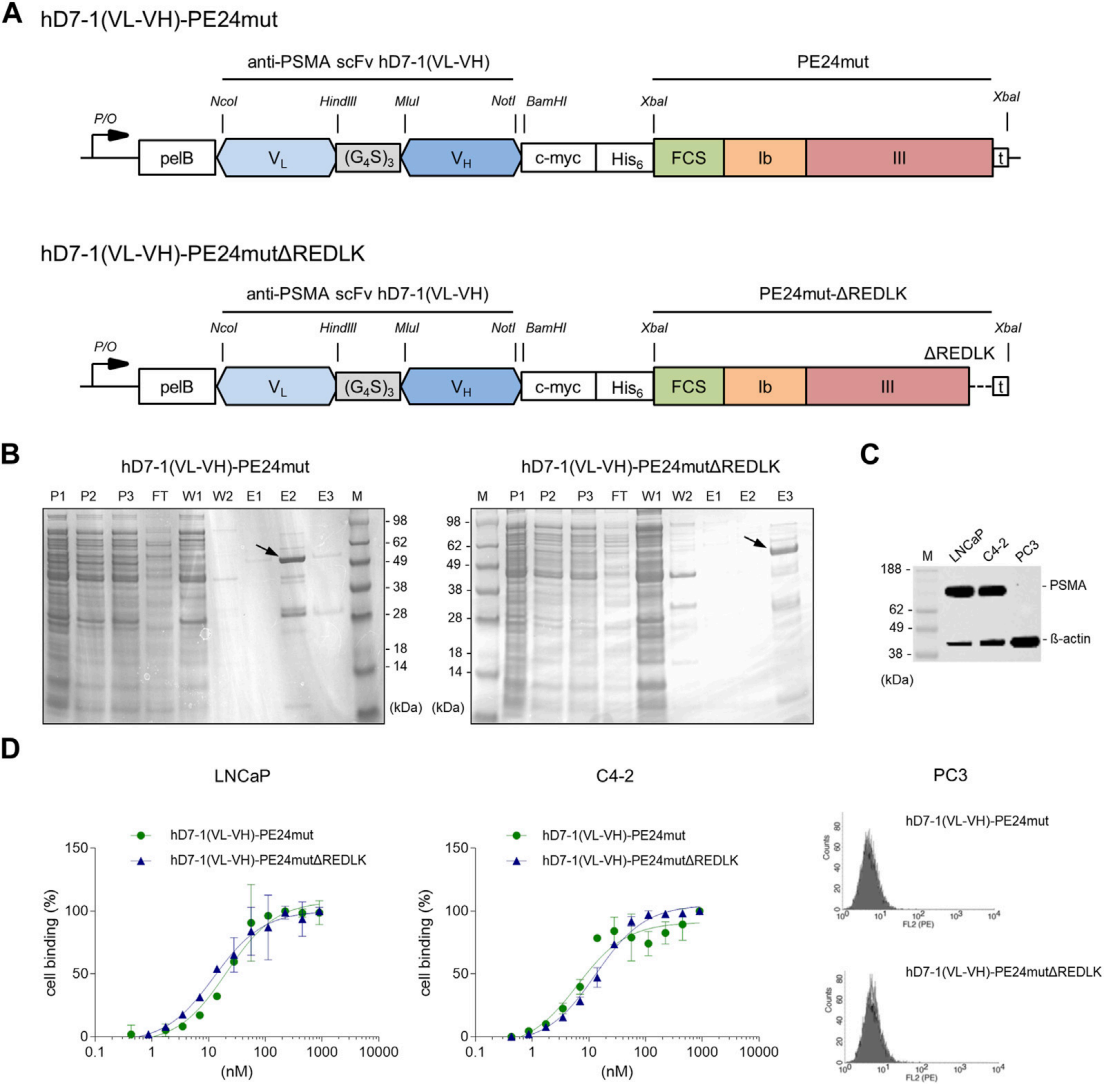
Characterization of the anti-PSMA immunotoxins. **(A)** Schematic representation of the immunotoxins hD7-1(VL-VH)-PE24mut and hD7-1(VL-VH)-PE24mutΔREDLK in the bacterial expression vector pHOG21. **(B)** SDS gel with immunotoxins (arrows) in the elution fractions after purification by IMAC with P1-3, periplasmatic fractions; FT, flow through; W1-2, wash fractions; E1-3 elution fractions; m, marker. **(C)** PSMA expression of prostate cancer cells as shown by Western blot. **(D)** Binding of the immunotoxins to PSMA-positive LNCaP and C4-2 cells and to PSMA-negative PC3 cells at saturation concentration as shown by flow cytometry. Abbreviations: c-myc, human c-myc tag; His_6_, hexahistidine tag; FCS, furin cleavable site; pelB, leader peptide for periplasmatic expression; V_H_, variable fragment of the heavy chain; V_L_, variable fragment of the light chain.

The anti-PSMA immunotoxins hD7-1(VL-VH)-PE24mut and hD7-1(VL-VH)-PE24mutΔREDLK (Figure 1A) were cloned in the vector pHOG21, periplasmatically expressed in *E. coli* and purified by IMAC. The immunotoxins were analyzed by SDS-PAGE. Both were found as about 56 kDa proteins in 48 or 62% purity, respectively in the elution fractions (Figure 1B).

PSMA expression in LNCaP and C4-2 cells was verified by Western blot analysis (Figure 1C). Binding of hD7-1(VL-VH)-PE24mut and hD7-1(VL-VH)-PE24mutΔREDLK to the prostate cancer cells was determined by flow cytometry. Comparable K_d_ values were calculated for LNCaP (K_d_ = 20.9 ± 3.9 nM and 12.6 ± 2.4 nM, respectively) and C4-2 cells (K_d_ = 10.6 + 1.6 nM and 13.4 ± 1.2 nM, respectively). No binding was found to PSMA-negative PC3 cells (Figure 1D). Thus, deletion of the REDLK motif did not lead to a change in binding affinity or specificity of the immunotoxin.

**FIGURE 2:**
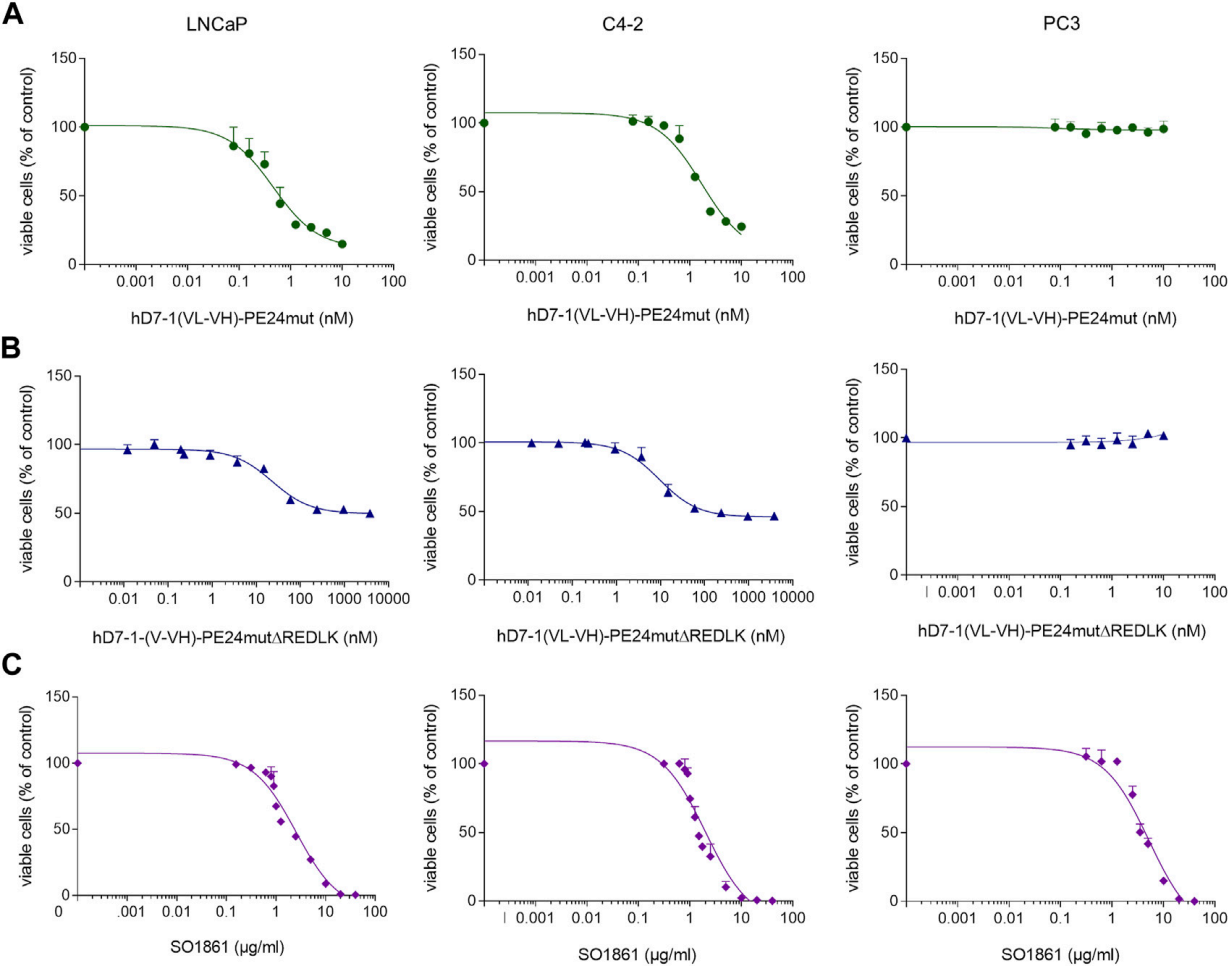
Cytotoxicity of the immunotoxins **(A)** hD7-1(VL-VH)-PE24mut, **(B)** hD7-1(VL-VH)-PE24mutΔREDLK and **(C)** SO1861 on prostate cancer cells as determined by WST viability assay.

The immunotoxin hD7-1(VL-VH)-PE24mut caused a specific and concentration-dependent cytotoxicity in PSMA-positive target cells. IC_50_ values of 0.74 ± 0.05 nM were reached on LNCaP cells and of 1.71 ± 0.36 nM on C4-2 cells, respectively, after 48 h incubation (Figure 2A). In comparison, the immunotoxin hD7-1(VL-VH)-PE24mutΔREDLK showed a 117- to 5,130-fold reduced cytotoxicity with IC_50_ values of 3800 nM and 200 nM in both cell lines, respectively (Figure 2B). No cytotoxicity of both immunotoxins was measured in PC3 cells (Figures 2A, B). This is consistent with observations that PE-based immunotoxins without REDLK motif can no longer enter the cytosol via the KDEL receptor-mediated transport pathway to inhibit protein biosynthesis and therefore show highly reduced cytotoxicity (Chaudhary et al., 1990). Additionally, we tested the cytotoxicity of SO1861, which is known to be cytotoxic at high concentrations by interference with the endosomal membranes after internalization (Thakur et al., 2013). IC_50_ values of 2.54 ± 0.34 μg/mL, 2.00 ± 0.39 μg/mL and 5.20 ± 0.96 μg/mL were determined on LNCaP, C4-2 and PC3 cells, respectively (Figure 2C).

In the next step we examined the effects of the immunotoxins in combination with SO1861 on the prostate cancer cells. First, we tested different concentrations of hD7-1(VL-VH)-PE24mut in combination with 1 μg/mL SO1861 on LNCaP cells and found no significantly enhanced cytotoxicity (IC_50_ = 0.70 ± 0.13 nM) compared to the immunotoxin alone (IC_50_ = 0.74 ± 0.05 nM) (Table 1; Supplementary Figure S1A). In contrast, cytotoxicity of hD7-1(VL-VH)-PE24mutΔREDLK in combination with different concentrations of SO1861 (0.625–2.5 μg/mL) was up to 380,000-fold enhanced in LNCaP cells (Table 1; Supplementary Figure S1B) and up to 6,666-fold enhanced in C4-2 cells (Table 1; Supplementary Figure S1C) compared to immunotoxin monotherapy. No enhanced cytotoxicity of hD7-1(VL-VH)-PE24mutΔREDLK in combination with the highest concentration of 2.5 mg/mL SO1861 was measured in PC3 control cells (Table 1; Supplementary Figure S1C). Instead, the combination caused an approx. 20% reduction in viable cells, which corresponds and can therefore be attributed to the cytotoxicity of 2.5 μg/mL SO1861 as a single substance (Table 1; Supplementary Figure S1C). Calculation of the CI revealed synergism of both components in a concentration range between 0.8 and 1.25 μg/mL SO1861 on LNCaP and between 0.9 and 1.25 μg/mL SO1861 on C4-2 cells (Table 1). Calculation of sensitivity (viability of PSMA-positive LNCaP or C4-2 cells) and specificity (viability of PSMA-negative PC3 cells) resulted in therapeutic windows with 75% sensitivity and 95% specificity of 0.96–1.49 μg/mL SO1861 on LNCaP (Supplementary Figures S2A, C) and of 1.18–1.48 μg/mL SO1861 on C4-2 cells (Supplementary Figures S2B, C), concentration ranges that reflected those synergistic concentration ranges that emerged when the CI were calculated.

**TABLE 1:** Cytotoxicity of the anti-PSMA immunotoxins in combination with SO1861 in prostate cancer cells. The IC_50_ values, defined as the immunotoxin concentrations that leads to a reduction in cell viability by 50% after 48 h, were calculated by WST test. Combination Index (CI) with <0.8, synergistic (syn); 0.8–1.2, additive (add); >1.2 antagonistic (anta). Enhancement of cytotoxicity (EOC) of the immunotoxins by SO1861 was calculated with help of the formula EOC = IC_50_ (Immunotoxin)/IC_50_ (Immunotoxin + SO 1861).

	LNCaP				C4-2				PC3			
	IC_50_ (nM)	CI*	Syner-gism*	EOC** (-fold)	IC_50_ (nM)	CI*	Syner-gism*	EOC** (-fold)	IC_50_ (nM)	CI*	Syner-gism*	EOC** (-fold)
hD7-1(VL-VH)-PE24mut	0.74	—	—	—	1.71	—	—	—	> 10	—	—	—
hD7-1(VL-VH)-PE24mut+ 1 μg/mL SO1861	0.7	1.784	anta	—	—	—	—	—	—	—	—	—
hD7-1(VL-VH)-PE24mutΔREDLK	3,800	—	—	1	200	—	—	1	> 10	—	—	—
hD7-1(VL-VH)-PE24mutΔREDLK+ 0.625 μg/mL SO1861	> 10	nd	nd	nd	—	—	—	—	—	—	—	—
hD7-1(VL-VH)-PE24mutΔREDLK+ 0.8 μg/mL SO1861	0.24	0.325	syn	15,833	> 10	nd	nd	nd	—	—	—	—
hD7-1(VL-VH)-PE24mutΔREDLK+ 0.9 μg/mL SO1861	0.19	0.365	syn	20,000	0.13	0.450	Syn	1,538	—	—	—	—
hD7-1(VL-VH)-PE24mutΔREDLK+ 1 μg/mL SO1861	0.05	0.406	syn	76,000	0.08	0.500	Syn	2,500	—	—	—	—
hD7-1(VL-VH)-PE24mutΔREDLK+ 1.25 μg/mL SO1861	0.02	0.508	syn	190,000	0.03	0.624	syn	6,667	—	—	—	—
hD7-1(VL-VH)-PE24mutΔREDLK+ 2.5 μg/mL SO1861	0.01	1.601	add	—	0.000003	1.249	anta	—	> 10	nd	nd	nd

**FIGURE 3:**
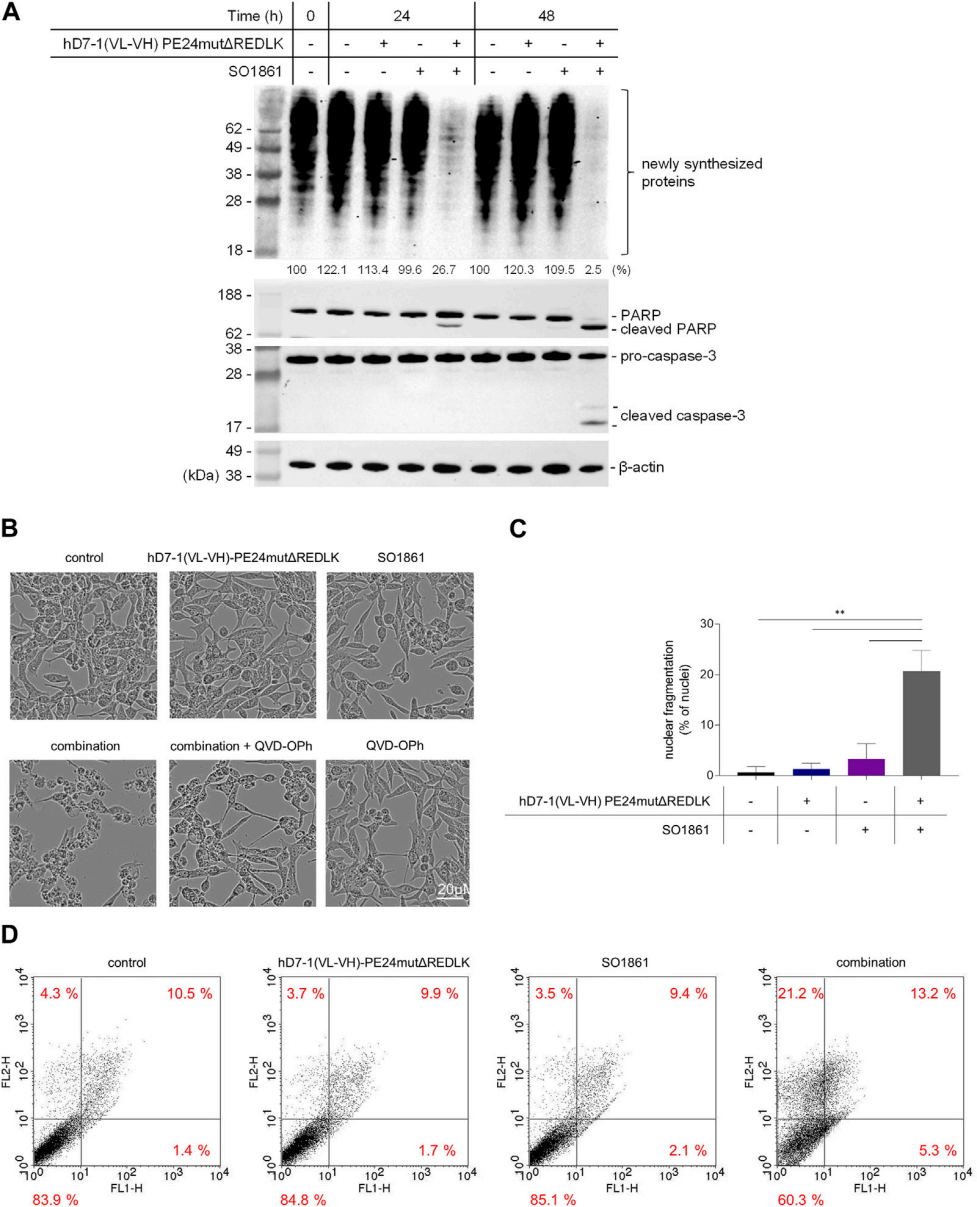
Inhibition of protein biosynthesis and induction of apoptosis and necrosis after treatment in LNCaP cells with 2.5 nM hD7-1(VL-VH)-PE24mutΔREDLK in combination with 1.25 μg/mL SO 1861. **(A)** Inhibition of protein biosynthesis as shown by puromycin Western blot analysis and induction of apoptosis as demonstrated by PARP and Caspase-3 cleavage after combination treatment. **(B)** Morphological characteristics of LNCaP cells after treatment with hD7-1(VL-VH)-PE24mutΔREDLK and SO1861 alone or in combination for 48 h. Apoptosis was prevented by addition of the pan caspase inhibitor QVD-OPh. **(C)** Nuclear fragmentation as hallmark of apoptosis was analyzed by fluorescence microscopy after Hoechst33342 staining. MW ± SD from 3 independent experiments. Student’s t-test (unpaired, parametric with Welch’s correction) with **p < 0.01. **(D)** AV (FL1-H) and EthD-III (FLH-2) staining was done after 48 h incubation to distinguish between living cells (AV^−^/EthD-III^-^), early apoptosis (AV^+^/EthD-III^-^), late apoptosis (AV^+^/EthD-III^+^) and necrosis (AV-/EthD-III^+^) and analyzed by flow cytometry.

Next, LNCaP cells were incubated with 2.5 nM hD7-1(VL-VH)-PE24mutΔREDLK and 1.25 μg/mL SO1861 alone or in combination for 24 and 48 h. Western Blot analysis revealed that protein biosynthesis of the LNCaP cells was reduced up to 26.7% after 24 h and up to 2.5% after 48 h by combination treatment. Inhibition of protein biosynthesis was accompanied by PARP cleavage and activation of Caspase-3 (Figure 3A). Morphological changes in the LNCaP cells after treatment with hD7-1(VL-VH)-PE24mutΔREDLK + SO1861 were marked by cell shrinkage, rounding and formation of apoptotic bodies and could be reversed in parts by addition of the *pan-*caspase inhibitor QVD-OPh (Figure 3B). Nuclear fragmentation was detected in 20.7% ± 4.2% cells after combination treatment, which was significantly higher compared to untreated cells (0.7% ± 1.2%) or cells treated with immunotoxin (1.3% ± 1.2%) or SO 1861 (3.3% ± 3.1%) monotherapies (Figure 3C). This underlined the observations from the Western Blots that apoptosis was induced after combination treatment. Moreover, Annexin V (AV) and Ethidium Homodimer III (EthD-III) staining was done to distinguish between living cells (AV^−^/EthD-III^-^), early apoptosis (AV^+^/EthD-III^-^), late apoptosis (AV^+^/EthD-III^+^) and necrosis (AV-/EthD-III^+^). As shown in Figure 3D, combination treatment led to 18.5% apoptotic cells (AV+/EthD-III^-^ and AV+/EthD-III^+^) after 48 h, compared to 11.5%–11.9% of apoptotic cells in samples, which were left untreated or which were treated with the monotherapies. At the same time the percentage of necrotic cells (AV-/EthD-III^+^) also increased from a basal level between 3.5% and 4.3% to 21.2%. Taken together, our cell death analyses revealed that the combination treatment led to protein biosynthesis inhibition followed by induction of apoptosis and necrosis in the prostate cancer cells.

**FIGURE 4:**
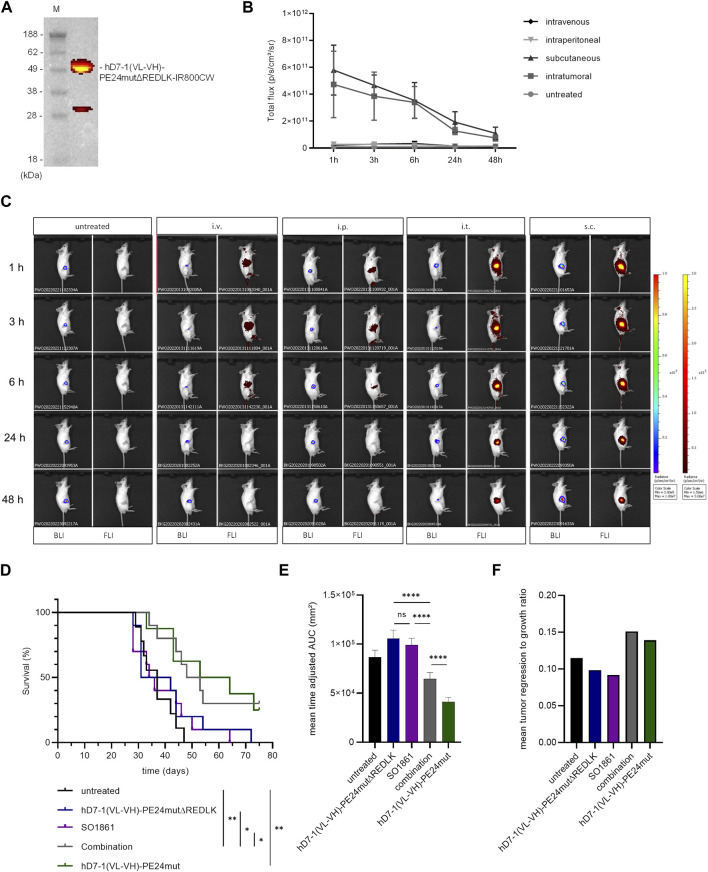
*In vivo* experiments. **(A)** The immunotoxin hD7-1(VL-VH)-PE24mutΔREDLK was labeled with the fluorescence dye IR800CW and analyzed on an SDS gel under fluorescence light. **(B)** Intratumoral fluorescence of hD7-1(VL-VH)-PE24mutΔREDLK-IR800CW in mice after intraveneous, intraperitoneal, subcutaneous or intratumoral injection. **(C)** Example images of mice bearing C4-2^luc+^ tumors as shown by BLI and biodistribution of hD7-1(VL-VH)-PE24mutΔREDLK-IR800CW (FLI) at different times after injection. **(D)** Survival of tumor bearing mice treated with hD7-1(VL-VH)-PE24mutΔREDLK in combination with SO1861 or with single drugs as determined by Kaplan-Meier analysis and log-rank test with *p < 0.05, **p < 0.01. **(E)** Inhibition of tumor growth in animals treated with combination therapy or single drugs as determined by time-adjusted AUC and Student’s *t-*test (unpaired, parametric with Welch’s correction) with ****p < 0.0001. **(F)** Mean tumor regression to growth ratio of animals treated with combination therapy or single drugs.

The *in vivo* activity of hD7-1(VL-VH)-PE24mutΔREDLK in combination with SO1861 was further examined in SCID mice with subcutaneous prostate tumor xenografts. Initially, we tested the uptake of hD7-1(VL-VH)-PE24mutΔREDLK by the tumor after injection via different routes. For this, the immunotoxin was labeled with the fluorescence dye IR800CW (Figure 4A). hD7-1(VL-VH)-PE24mutΔREDLK-IR800CW was injected i.v., i.p., i.t. and s.c. into the tumor bearing animals. Fluorescence Imaging (FLI) for the detection of the immunotoxin and simultaneous Bioluminescence Imaging (BLI) for the visualization of the tumors were done. As shown in Figures 4B, C, highest uptake of hD7-1(VL-VH)-PE24mutΔREDLK-IR800CW by the tumor was found 1 h after s.c. injection, which was comparable to that after i.t. injection. Only low uptake was found after i.v. or i.p injection. After 1 h a decrease of the intratumoral immunotoxin was detected in all animals. For the following experiments, we therefore decided to inject the immunotoxin s.c. followed by s.c. injection of SO1861 1 h later.

For therapy, mice bearing tumors with 60–80 mm³ in size were injected with hD7-1(VL-VH)-PE24mutΔREDLK and SO1861 alone or in combination on days 1, 3, 8, 10, 15 and 17 of treatment. Control animals were treated with hD7-1(VL-VH)-PE24mut or left untreated. Tumor volume was measured until end of treatment on day 75. Animals that fulfilled one of the termination criteria were euthanized. Mice treated with the combination showed significantly enhanced mean overall survival (50.5 days) compared to mice treated with the immunotoxin alone (36.5 days), with SO1861 alone (35.0 days) or that remained untreated (37.0 days). Treatment of animals with hD7-1(VL-VH)-PE24mut resulted in a mean overall survival of 58.5 days (Figure 4D). 1/10 animals treated with combination and 2/8 animals treated with hD7-1(VL-VH)-PE24mut showed complete tumor remission until end of treatment. Enhanced overall survival of the combination group was accompanied by significant inhibition of tumor growth (Figure 4E) and by an enhanced tumor to regression ratio (Figure 4F). No significant weight loss of more than 20% as sign of toxicity was measured in any animal during treatment, underlining the high safety of our therapeutic approach (Supplementary Figure S3).

## 4 Discussion

In the last decades PE-based immunotoxins were developed against a variety of tumors and have successfully entered the clinic. Main dose-limiting adverse side effects comprise immune responses against non-human/humanized parts of the immunotoxins, evocation of VLS and the uptake of the immunotoxins by normal cells due to non-tumor specific target antigen expression (Havaei et al., 2021). For the construction of the immunotoxin hD7-1(VL-VH)-PE24mut we used a humanized scFv as binding domain and PE24mut as toxin domain, which are expected to have a reduced immunogenicity and lower risk of developing VLS in future clinical use (Weldon et al., 2013; Mazor and Pastan, 2020). Although PSMA is highly organ specific, it cannot be excluded that normal tissues with PSMA expression (i.e., kidney, salivary glands, brush border of the duodenal columnar epithelium) could also be damaged by the immunotoxin.

The C-terminal KDEL-like sequence REDLK of PE was identified to be essential for the retrograde transport of the toxin from the sorting endosomes via the KDEL-receptor mediated pathway through the Golgi apparatus into the ER, from which it is transported into the cytosol. Indeed, deletion of the REDLK sequence in PE-based immunotoxins resulted in a more than 1000-fold reduction of cytotoxicity (Chaudhary et al., 1990). In other studies, the REDLK sequence was replaced by KDEL in targeted toxins consisting of PE40 and either TGF-α or hIL2R as binding domain. With this mutation, increased cytotoxicity in different cell lines, but also an enhanced toxicity in mice were observed (Seetharam et al., 1991).

We deleted the REDLK sequence of the anti-PSMA immunotoxin to avoid KDEL-receptor mediated routing with proteasomal degradation and to enhance endolysosomal entrapment. REDLK deletion did not influence the binding affinity of the immunotoxin, however showed an up to 5,130-fold reduced cytotoxicity on the prostate cancer cells compared to the immunotoxin with the REDLK sequence. This is in line with studies of Chaudhary et al., where the KDEL receptor mediated pathway was identified as the main route of the toxic domain of PE to reach the cytosol (Chaudhary et al., 1990).

By addition of subtoxic doses of SO1861 we measured an up to 190,000-fold enhanced cytotoxicity of hD7-1(VL-VH)-PE24mutΔREDLK, which was also up to 57-fold enhanced compared to the immunotoxin with the REDLK sequence. Cytotoxicity was still based on protein biosynthesis inhibition and induction of apoptosis, which underlines the natural effects of the toxin domain on the ribosome after release into the cytosol. In contrast, the cytotoxicity of hD7-1(VL-VH)-PE24mut was not enhanced by SO1861, which is in line with the observations of Weng and colleagues, who found no enhanced cytotoxicity of an anti-EGFR targeted toxin containing the PE toxin domain after co-incubation with the endosomal escape enhancer SA1641 (Weng et al., 2012a). Our observations prove that lysosomal and/or proteasomal degradation of PE-based immunotoxins has high significance in the loss of their cytotoxic activity during cell routing via the KDEL receptor mediated pathway.

We measured a higher decrease in cytotoxicity of our immunotoxin hD7-1(VL-VH)-PE24mut by REDLK deletion and in turn a larger increase in cytotoxicity after addition of SO1861 in LNCaP cells than in C4-2 cells. Bachran and colleagues found also different enhancement factors between 2,800 and 2,500,000 in EGFR-expressing cell lines after addition of saponins to a targeted toxin consisting of epidermal growth factor (EGF) and the plant toxin saporin-3. Consistent with our study, higher enhancement factors were reached in cells, in which a higher IC_50_ value of the targeted toxin without saponin were achieved (Bachran et al., 2010). The reasons why different cell lines react so differently to enhanced endosomal escape, needs to be analyzed in more detail. They might include different receptor expression rates, internalization rates, intracellular transport routes, degradation rates, endosomal leakage, proteasome activities, and natural competition and inhibitory reactions.

**FIGURE 5:**
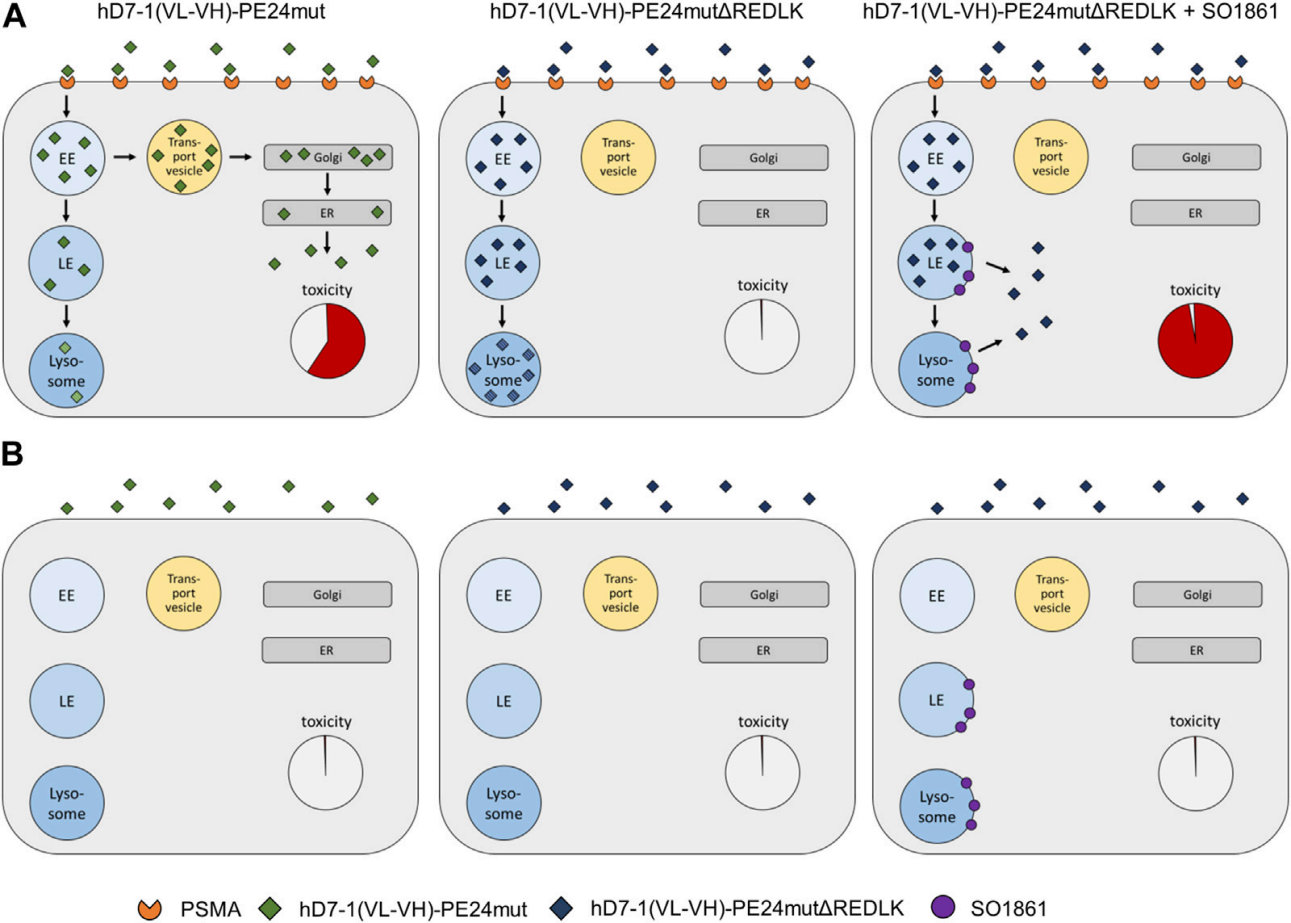
Schematic representation of endosomal escape and cytotoxicity of PE-based immunotoxins. **(A)** Endosomal escape and toxicity in PSMA expressing prostate cancer cells. **(B)** Endosomal escape in PSMA-negative cells. Abbreviations: EE, early endosome; ER, endoplasmic reticulum; LE, late endosome; Golgi, Golgi network.

It is clearly established that glycosylated triterpenoids act as very strong endosomal escape enhancers for some proteins but not at all for others (Fuchs et al., 2017). So far, it has only been possible to speculate about the reason. With the present work, we prove for the first time that intracellular trafficking is highly important for the effect of endosomal escape enhancers and that rerouting of proteins can make unaffected proteins accessible to the enhancer effect. Future experiments investigating the cell routing of the immunotoxin and its interaction with SO1861 should demonstrate the presumed release from the endolysosomes into the cytosol at the molecular level. Fluorescence microscopy has previously been used to examine the impact of glycosylated triterpenoids on the endosomal escape of ribosome-inactivating proteins. Weng et al. assessed the effect of SA1641 on the intracellular distribution of the ribosome-inactivating protein saporin (Weng et al., 2012b) and the impact of SO1861 on the endosomal escape of dianthin was investigated by Gilabert-Oriol et al. (Gilabert-Oriol et al., 2015). These publications visually support the endosomal escape enhancement and release into the cytosol mediated by glycosylated triterpenoids. Together with the present work on rerouting, we now have a much better idea of how endosomal escape enhancers work (Figure 5).

Cell death induced by our treatment was characterized by induction of apoptosis, which is well described after intoxication of cells with PE or PE-based immunotoxins (Du et al., 2010). In addition to apoptosis, we also found evidence that necrosis was induced with our combination therapy. It is known that cells that were, e.g., treated by UV or γ-irradiation undergo apoptosis, but can also show signs of necrosis. Necrosis is a type of immunogenic cell death (ICD), because necrotic cells are able to induce antigen-specific and particularly CD8^+^ T-cell mediated adaptive immune responses (Gamrekelashvili et al., 2015). In former experiments signs of necrosis and release of inflammatory mediators were also measured in tumors after treatment with PE-based immunotoxins (Luther et al., 2010; Leshem et al., 2018) and simultaneous treatment of mice bearing mesothelioma tumors with the anti-mesothelin immunotoxin SS1P and an checkpoint inhibitor against the Cytotoxic T-Lymphocyte-Associated Protein 4 (CTLA-4) resulted in enhanced antitumor activity compared to monotherapies and in protection from tumor recurrence (Leshem et al., 2018). Future experiments will show whether the induction of necrosis by our therapy leads to ICD in prostate cancer cells and whether this will be beneficial for an improved systemic tumor response, specifically with regard to the damage of tumor cells that were not reached by our drugs or that are apoptosis-resistant and with regard to a longer lasting memory T cell response. Treatment of mice bearing prostate tumor xenografts with hD7-1(VL-VH)-PE24mutΔREDLK and SO1861 resulted in significantly enhanced survival and inhibition of tumor growth compared to mice treated with the monotherapies. In contrast to the cell culture experiments where the combination therapy was superior, the antitumor effects of hD7-1(VL-VH)-PE24mut were not fully achieved with this treatment in mice. It is obvious that the effect can only take place when both substances are inside the endosomes at the same time. This is always the case in cell culture because the substances are continuously present. *In vivo*, pharmacokinetic effects, in particular distribution, metabolism, and excretion play an important role. Therefore, we here used sc injection, which is not optimal but better to partially compensate for kinetic effects as the toxin remains longer in the proximity of the tumor, which increases the probability for co-existence in endosomes. In the past, we already showed that iv injection is also possible (Panjideh et al., 2022). However, this requires even more efforts to find the balance for the different kinetics.

Since our two-component system is difficult to transfer to patients, future research is focused on the development of a one-component system consisting of the modified immunotoxin and SO1861 (Postel and Fuchs, 2019). It is expected that a one-component system, in which divergent kinetics no longer play a role, will show superior behavior *in vivo*, could lead to reduced drug doses with diminished side effects and would be suitable for clinical studies.

## 5 Conclusion

We showed for the first time that detoxification of an PE-based anti-PSMA immunotoxin by REDKL deletion and retoxification by addition of the endosomal escape enhancer SO1861 resulted in enhanced cytotoxicity. Our study will help to understand the interplay of PE and other proteins with similar intracellular trafficking and endosomal escape enhancers in future.

Since both components exhibit different pharmacokinetics and metabolism, a major challenge will be to transfer the approach into the clinic. A one-component system consisting of both drugs could lead to reduced doses with maintenance of antitumor efficacy and reduced side effects in future.

## Data Availability

The original contributions presented in the study are included in the article/Supplementary Material, further inquiries can be directed to the corresponding author.
