# Supplementary material for: Enhanced cytotoxicity of a Pseudomonas Exotoxin A based immunotoxin against prostate cancer by addition of the endosomal escape enhancer SO1861
Source: Front Pharmacol. 2023 Jul 6;14:1211824. doi: 10.3389/fphar.2023.1211824 (PMC10358361; doi:10.3389/fphar.2023.1211824)
Supplement: Supplementary file 1 [file Presentation1.PPTX]

## Slide 1
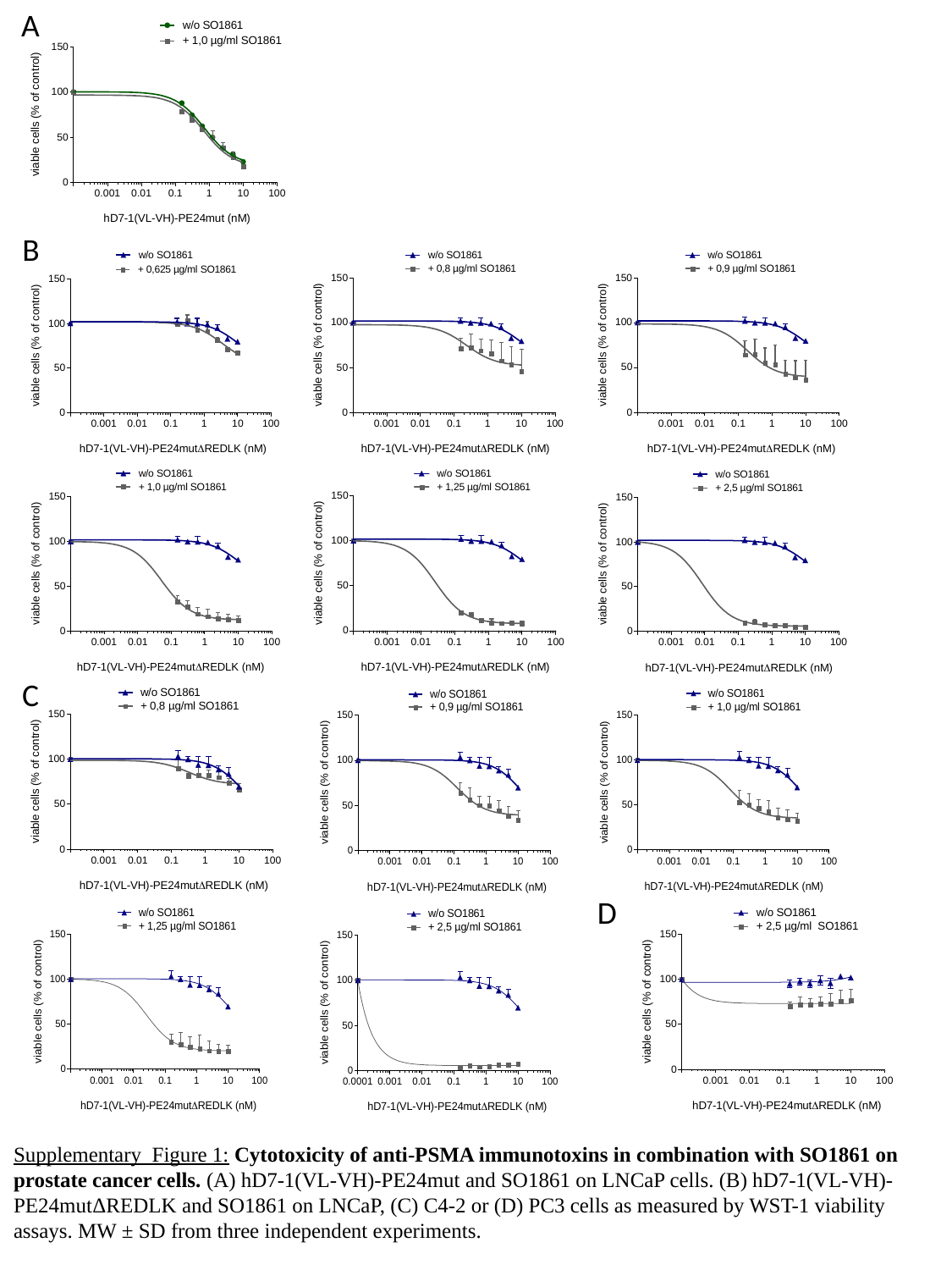

A
B
C
D
Supplementary Figure 1: Cytotoxicity of anti-PSMA immunotoxins in combination with SO1861 on prostate cancer cells. (A) hD7-1(VL-VH)-PE24mut and SO1861 on LNCaP cells. (B) hD7-1(VL-VH)-PE24mutΔREDLK and SO1861 on LNCaP, (C) C4-2 or (D) PC3 cells as measured by WST-1 viability assays. MW ± SD from three independent experiments.

## Slide 2
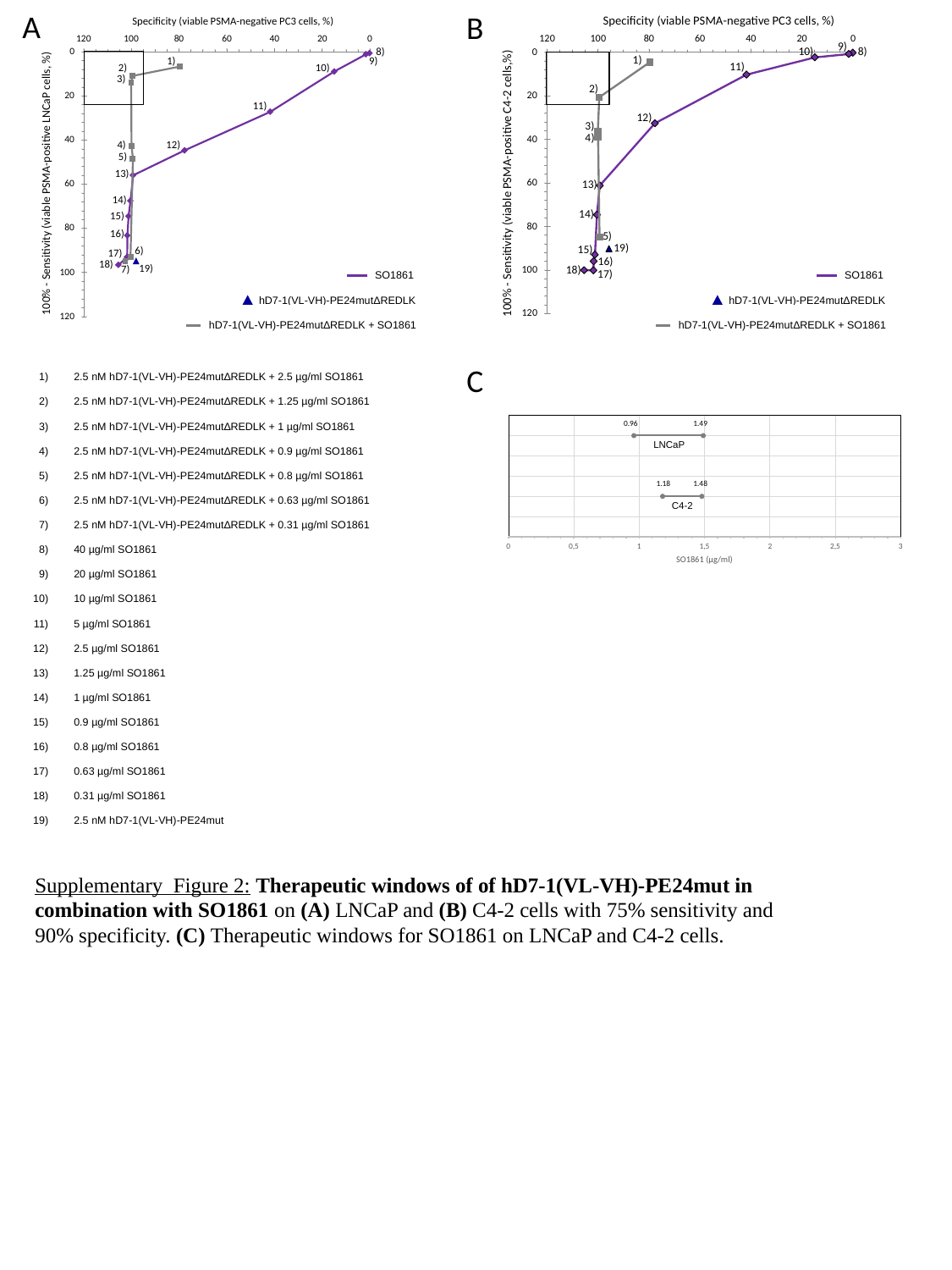

A
B
SO1861
SO1861
hD7-1(VL-VH)-PE24mutΔREDLK
hD7-1(VL-VH)-PE24mutΔREDLK
hD7-1(VL-VH)-PE24mutΔREDLK + SO1861
hD7-1(VL-VH)-PE24mutΔREDLK + SO1861
C
| 1) | 2.5 nM hD7-1(VL-VH)-PE24mutΔREDLK + 2.5 µg/ml SO1861 |
| --- | --- |
| 2) | 2.5 nM hD7-1(VL-VH)-PE24mutΔREDLK + 1.25 µg/ml SO1861 |
| 3) | 2.5 nM hD7-1(VL-VH)-PE24mutΔREDLK + 1 µg/ml SO1861 |
| 4) | 2.5 nM hD7-1(VL-VH)-PE24mutΔREDLK + 0.9 µg/ml SO1861 |
| 5) | 2.5 nM hD7-1(VL-VH)-PE24mutΔREDLK + 0.8 µg/ml SO1861 |
| 6) | 2.5 nM hD7-1(VL-VH)-PE24mutΔREDLK + 0.63 µg/ml SO1861 |
| 7) | 2.5 nM hD7-1(VL-VH)-PE24mutΔREDLK + 0.31 µg/ml SO1861 |
| 8) | 40 µg/ml SO1861 |
| 9) | 20 µg/ml SO1861 |
| 10) | 10 µg/ml SO1861 |
| 11) | 5 µg/ml SO1861 |
| 12) | 2.5 µg/ml SO1861 |
| 13) | 1.25 µg/ml SO1861 |
| 14) | 1 µg/ml SO1861 |
| 15) | 0.9 µg/ml SO1861 |
| 16) | 0.8 µg/ml SO1861 |
| 17) | 0.63 µg/ml SO1861 |
| 18) | 0.31 µg/ml SO1861 |
| 19) | 2.5 nM hD7-1(VL-VH)-PE24mut |
Supplementary Figure 2: Therapeutic windows of of hD7-1(VL-VH)-PE24mut in combination with SO1861 on (A) LNCaP and (B) C4-2 cells with 75% sensitivity and 90% specificity. (C) Therapeutic windows for SO1861 on LNCaP and C4-2 cells.

## Slide 3
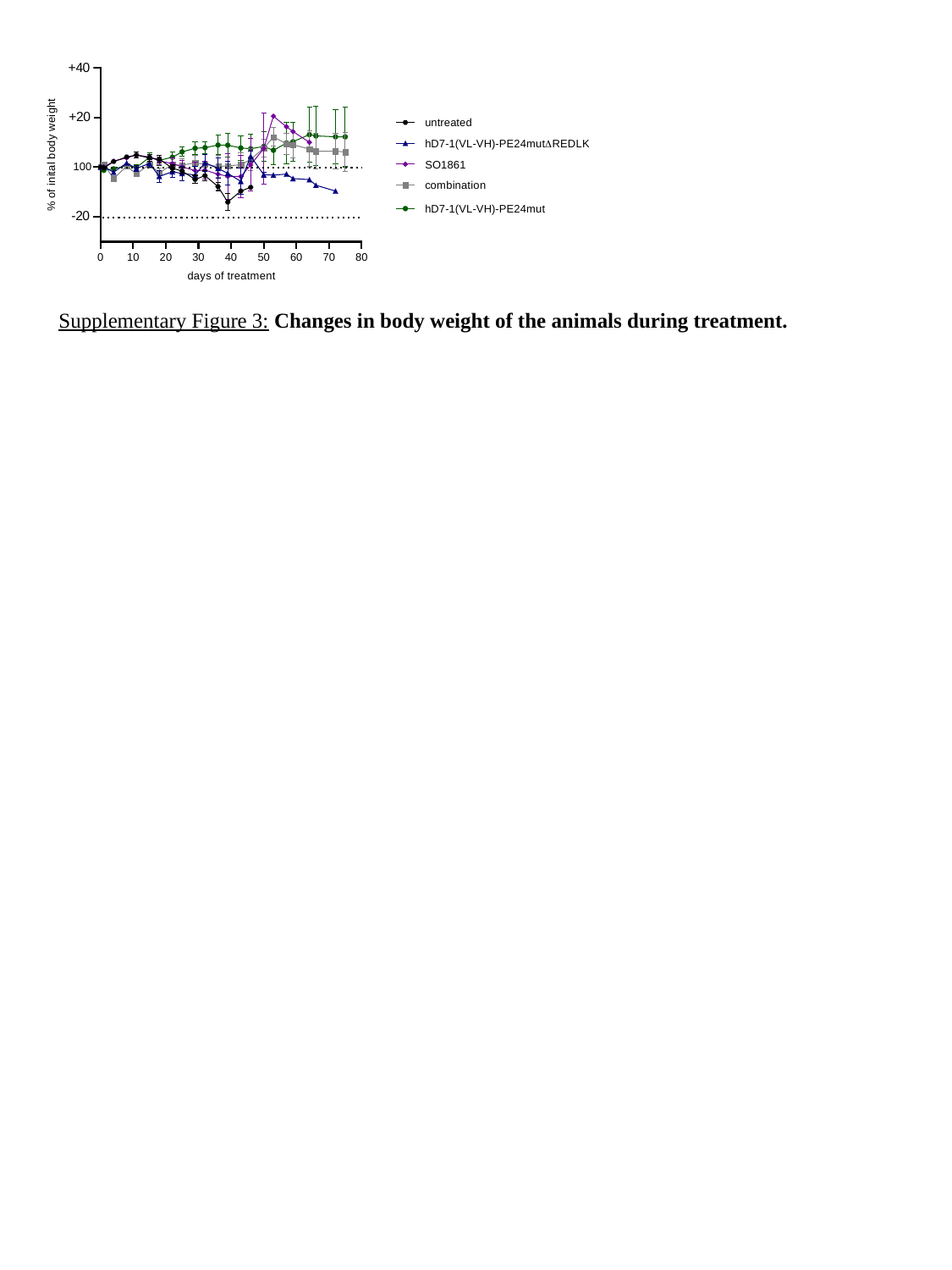

Supplementary Figure 3: Changes in body weight of the animals during treatment.
